# The Osteosarcoma Tumor Microenvironment: From Cellular Interactions to Advanced Preclinical Models

**DOI:** 10.3390/cancers18182909

**Published:** 2026-09-09

**Authors:** Giuseppe Di Feo, Alessandra Di Paola, Oriana Di Domenico, Lucia Argenziano, Elvira Pota, Daniela Di Pinto, Martina Di Martino, Maria Maddalena Marrapodi, Francesca Rossi

**Affiliations:** 1 Department of Woman, Child and General and Specialist Surgery, University of Campania “Luigi Vanvitelli”, 80138 Naples, Italy; giuseppe.difeo.bio@gmail.com (G.D.F.); a.dipaola@unilink.it (A.D.P.); didomenico.oriana.odd@gmail.com (O.D.D.); lucia.argenziano@unicampania.it (L.A.); elvira.pota@gmail.com (E.P.); daniela.dipinto@policliniconapoli.it (D.D.P.); martina.dimartino@policliniconapoli.it (M.D.M.); mariamaddalena.marrapodi@unicampania.it (M.M.M.); 2 Department of Life Sciences, Health and Health Professions, Link Campus University, 00165 Rome, Italy

**Keywords:** osteosarcoma, tumor microenvironment, organoids, 3D culture, bioprinting, patients derived xenografts

## Abstract

OS is the most common primary malignant bone tumor, predominantly affecting children, adolescents, and young adults. Despite advances in multimodal treatment, patient outcomes have remained largely unchanged over the past decades, particularly in cases of metastatic or recurrent disease. Increasing evidence highlights the pivotal role of the tumor microenvironment (TME) in OS progression, metastasis, immune evasion, and therapeutic resistance. The OS TME comprises a complex and dynamic network of tumor cells, immune cells, cancer-associated fibroblasts, mesenchymal stromal cells, endothelial cells, extracellular matrix components, and soluble factors that collectively regulate tumor behavior. This review summarizes the current understanding of the OS TME, highlighting the interactions between malignant cells and their surrounding microenvironment and their contribution to tumor growth, angiogenesis, invasion, metastatic dissemination, and treatment response. Moreover, here we discuss the strengths and limitations of the most relevant preclinical models currently used to investigate OS biology and evaluate novel therapeutic strategies. Three-dimensional (3D) culture systems, including spheroids, organoids, and biomimetic scaffold-based models, today are considered the models able to best recapitulate the structural, mechanical, and cellular complexity of the native tumor microenvironment than conventional two-dimensional cultures. In addition, patient-derived xenograft (PDX) models are reviewed for their ability to preserve the histopathological, molecular, and genetic characteristics of the original tumors, making them valuable platforms for translational research, biomarker discovery, and personalized medicine.

## Simple Summary

Osteosarcoma (OS) is a rare and aggressive bone cancer that mainly affects children and adolescents. Developing new and effective treatments is challenging because OS is a complex disease that interacts with the tumor microenvironment. Preclinical models are essential for studying tumor biology, growth, metastasis, and responses to therapy before treatments are tested in patients. Several models, including 3D cell cultures, organoids, and animal models, are used to reproduce different aspects of OS. However, each model has specific advantages and limitations, and no single model can completely reproduce the complexity of the tumor. The tumor microenvironment, which includes immune cells, bone cells, blood vessels, and an extracellular matrix, plays an important role in tumor progression and treatment resistance. This review summarizes the main preclinical models from spheroids to PDXs used to study OS and their ability to reproduce the tumor microenvironment, highlighting their importance for improving research and developing more effective therapies.

## 1. Introduction

OS is the most common and aggressive primary malignant bone tumor. It exhibits a bimodal age distribution, with a first incidence peak occurring in children and adolescents as a primary bone malignancy, and a second peak in older adults, where it often develops as a secondary malignancy associated with prior radiotherapy or underlying bone disorders. OS predominantly arises in the metaphyseal regions of long bones of the lower extremities and is characterized by a high propensity for local invasion and distant metastasis, particularly to the lungs [1,2]. Indeed, approximately 20% of patients present with metastatic disease at diagnosis, and nearly 90% of these metastases involve the lungs. Although OS accounts for only about 5% of pediatric malignancies, its aggressive biological behavior and early metastatic potential contribute to a high rate of cancer-related mortality [1]. The five-year survival rate is approximately 60–70% for patients with localized disease and less than 20% in those with metastatic OS. Furthermore, chemotherapy can dramatically affect bone metabolism, leading to osteoporosis and reduced bone mineral density, thereby increasing the risk of fractures [3,4]. A defining characteristic of OS is its highly immunosuppressive TME, which plays a critical role in tumor progression, metastasis, and therapeutic resistance. The TME consists of a complex and dynamic network of non-malignant cellular and non-cellular components, including immune cells, like CD4+,CD8+ T cells, and T regulatory cells (T-regs), fibroblasts, endothelial cells, the extracellular matrix (ECM), and soluble factors such as cytokines, chemokines, and metabolites [5]. Together, these elements establish a specialized niche that influences tumor growth and drug response. In OS, the TME is characterized by limited infiltration of cytotoxic T lymphocytes and the predominance of immunosuppressive signals, which promote immune evasion and contribute to the poor response to both conventional therapies and emerging immunotherapeutic approaches [6]. Notably, immune cells within the OS microenvironment can exert either antitumor or protumor activities depending on their phenotype and activation status, highlighting the importance of elucidating the molecular mechanisms of these interactions to identify more effective therapeutic strategies [7]. Another fundamental component of the OS microenvironment is represented by the ECM, which not only represents a structural scaffold made of proteins and polysaccharides, but actively regulates cell adhesion, migration, proliferation, and intracellular signaling, thereby facilitating tumor growth and metastatic dissemination [8]. Moreover, the hypoxic conditions that characterize the OS microenvironment further promote tumorigenesis by enhancing angiogenesis, modulating cytokine expression, suppressing normal cellular functions, and creating a permissive environment for tumor progression and distant metastasis [9,10].

## 2. Tumor Microenvironment in OS

**Figure 1:**
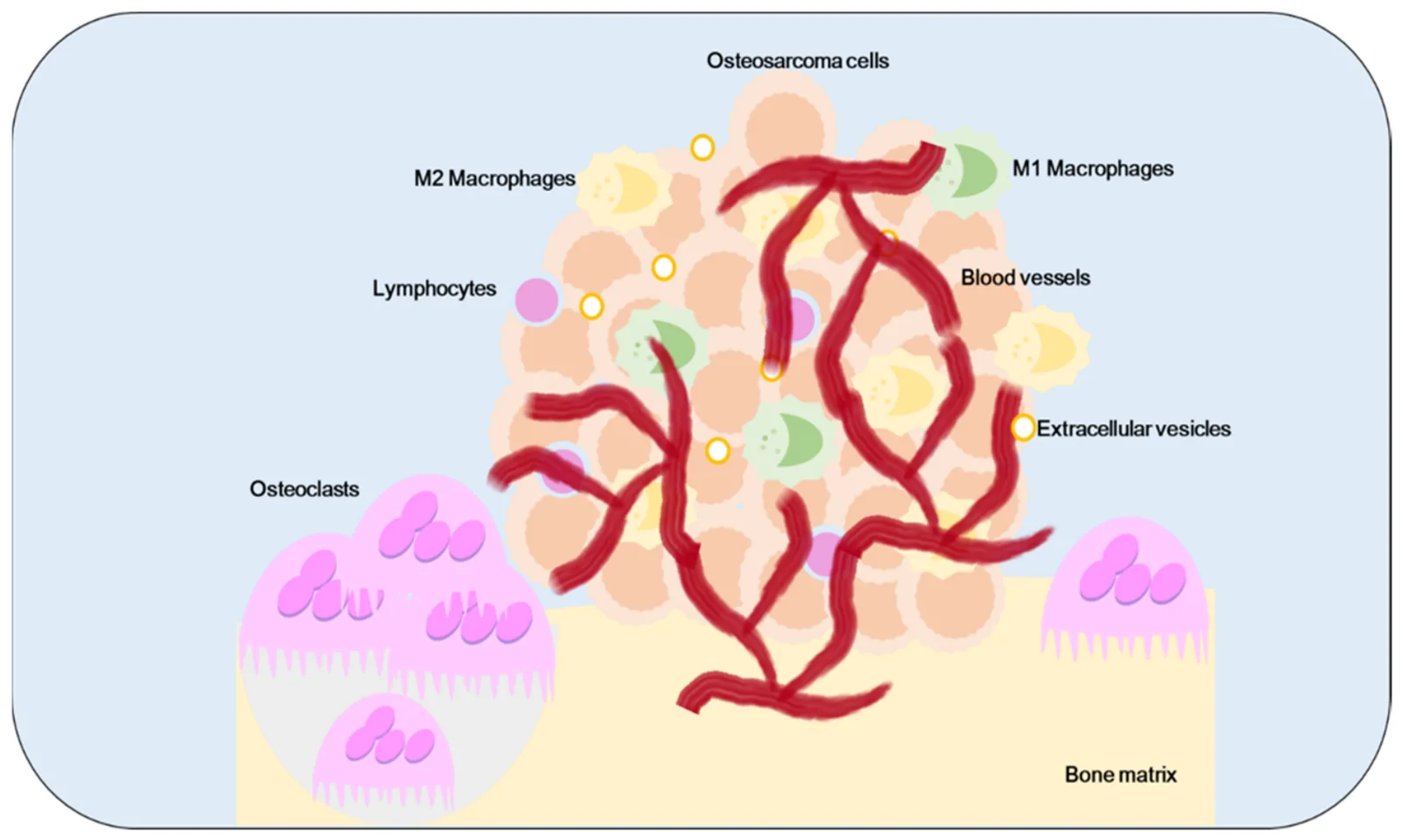
Schematic representation of the OS TME, highlighting the complex interplay between tumor cells and the surrounding stromal and immune components. The TME comprises osteoclasts, the bone matrix, blood vessels, extracellular vesicles, M1 and M2 macrophages, and lymphocytes, all of which contribute to tumor progression, immune modulation, angiogenesis, bone remodeling, and therapeutic response.

The unique composition and functional architecture of bone tissue have driven increasing interest in OS TME. In particular, there are investigations on both bone-resident and immune cell populations within the OS microenvironment but also emerging areas of research like extracellular vesicles (EVs) released from tumor cells (Figure 1).

### 2.1. Bone Cells

Osteoblasts, osteocytes, and osteoclasts constitute the components of the TME. OS primarily arises in the central regions of long bones and is characterized by abnormal osteoid formation and dysregulated bone remodeling. Based on histological and cellular features, it can be classified into three major subtypes: osteoblastic, chondroblastic, and fibroblastic.

#### 2.1.1. Osteoblasts

Among the major cellular components, osteoblasts play a crucial role in both the initiation and metastatic progression of OS [11,12]. Osteoblastic OS represents one of the most prevalent subtypes in both primary and metastatic disease. Within the OS TME, osteoblasts contribute to tumor progression by modulating osteogenic signaling pathways and promoting the expression of differentiation-related markers through the secretion of cytokines, including interleukin -6 (IL-6). The transcription factor RUNX family transcription factor 2 (RUNX2), a key regulator of osteoblast differentiation and maturation, controls osteoblast proliferation and cell-cycle progression and is therefore implicated in OS development and progression [13]. Beyond their physiological role in bone matrix formation, osteoblasts actively participate in extracellular matrix remodeling by secreting proteolytic enzymes, including matrix metalloproteinases (MMPs), and by interacting with osteoclasts during tumor-induced bone destruction. In several OS subtypes, osteoblast–osteoclast crosstalk contributes to the establishment of a pathological bone remodeling cycle. Before osteoclast-mediated degradation of the mineralized bone matrix, endosteal osteoblasts modify the unmineralized osteoid layer through the release of proteolytic enzymes, facilitating bone resorption [14,15]. Surface-associated MMPs expressed by osteoblasts further regulate this malignant remodeling process.

#### 2.1.2. Osteoclasts

Osteoclast-mediated bone degradation is a critical component of both primary and metastatic OS progression. Osteoclasts, derived from the monocyte–macrophage lineage, regulate bone resorption through complex interactions involving transcription factors, cytokines, and immune cells. A key regulator of osteoclast differentiation is the RANK–RANKL signaling pathway, which is frequently dysregulated in bone tumors. Activated osteoclasts release growth factors, including TGF-β, within resorption sites, promoting tumor cell proliferation and establishing a vicious cycle between bone destruction and tumor progression [16,17,18,19,20]. The interaction between OS cells and osteoclasts remains complex and context-dependent. While several studies demonstrate that OS cells can enhance osteoclast differentiation and resorptive activity, others report reduced osteoclast numbers in patient-derived OS samples, particularly in metastatic disease, suggesting that osteoclast regulation may vary according to tumor subtype and disease stage [21,22].

### 2.2. Immune Cells

The immune microenvironment represents an emerging perspective for understanding OS biology, with its main characteristic being an immunosuppressive state that enables tumor cells to evade immune surveillance. The immune components of the OS microenvironment can be broadly classified into cellular and acellular elements. Cellular components include various immune populations, such as tumor-associated macrophages (TAMs), tumor-associated neutrophils (TANs), myeloid-derived suppressor cells (MDSCs), mast cells, T cells, B cells, natural killer (NK) cells, and dendritic cells (DCs). In addition, non-immune cells, including mesenchymal stem cells (MSCs) and circulating tumor cells (CTCs), can interact with immune components and contribute to the establishment of immunosuppressive networks within the tumor microenvironment. Among the acellular factors, the complement system and EVs, particularly exosomes with immunomodulatory properties, have emerged as important regulators of OS immune responses and are currently under extensive investigation [7].

#### 2.2.1. Tumor-Associated Macrophages

TAMs represent one of the most abundant immune cell populations within the OS TME, accounting for a substantial proportion of the tumor mass [23]. They are involved in several processes that regulate tumor progression, including extracellular matrix remodeling, inflammation, angiogenesis, immune modulation, and tissue homeostasis. TAMs exhibit remarkable functional plasticity and are commonly classified into two major phenotypes: the pro-inflammatory M1 phenotype and the alternatively activated M2 phenotype. M1 macrophages generally contribute to antitumor immune responses and may limit metastatic progression, whereas M2 macrophages are associated with tissue repair, immunosuppression, and tumor-promoting activities, including enhanced invasion and metastasis [24]. The functional polarization of TAMs is regulated by multiple molecular mechanisms, including genetic alterations, dysregulation of signaling pathways such as NOTCH, macrophage polarization signals, and imbalances in T helper cell (Th1/Th2)-derived cytokine profiles [24,25,26,27]. Consequently, increasing efforts have focused on targeting TAM polarization, particularly by inhibiting M2-like macrophage activation, as a potential strategy to suppress OS progression and improve therapeutic outcomes. Although tumor-associated macrophages (TAMs) in OS have traditionally been classified according to the M1/M2 paradigm, increasing evidence indicates that this binary model does not adequately capture the transcriptional and functional heterogeneity of macrophages within the osteosarcoma microenvironment. Single-cell RNA sequencing studies have identified multiple TAM populations characterized by distinct molecular programs, suggesting that macrophage states exist along a dynamic continuum rather than as two mutually exclusive phenotypes [7,28]. In particular, three major TAM populations, referred to as M1-, M2-, and M3-TAMs, have been identified in osteosarcoma lesions. While M2-TAMs display expression of canonical immunosuppressive markers such as CD163, MRC1, MS4A4A, and MAF, M3-TAMs are characterized by FABP4 expression and have been associated with pulmonary metastatic dissemination, suggesting a potential link between macrophage metabolic states and metastatic progression [28]. More detailed single-cell analyses have further resolved the macrophage compartment into distinct transcriptional populations, including FABP5^+^, NR4A3^+^, TXNIP^+^, and IFIT1^+^ macrophages [7]. FABP5^+^ macrophages exhibit a transcriptional program enriched in lipid-metabolism genes, including APOC1, APOE, LGMN, and FABP5, whereas TXNIP^+^ macrophages show a more immunosuppressive phenotype characterized by MERTK, MRC1, STAB1, and CD163 expression [7]. Conversely, IFIT1^+^ macrophages display a prominent type I interferon and pro-inflammatory signature, with increased expression of CCL2, CCL3, CCL4, CXCL9, CXCL10, and TNF, together with enhanced expression of antigen-presentation machinery. Collectively, these findings support a model in which OS TAMs comprise a highly plastic and metabolically diverse population whose functional state is determined by the local tumor microenvironment, treatment history, and stage of disease. Importantly, defining these macrophage states at the molecular and spatial level may be more informative than conventional M1/M2 classification and could facilitate the development of macrophage-directed therapeutic strategies.

#### 2.2.2. Tumor-Associated Neutrophils

Neutrophils found within the tumor immune microenvironment, referred to as tumor-associated neutrophils (TANs), display a wide range of phenotypes and functions [29]. In OS, however, studies investigating TANs remain limited and are still in an early phase. When exposed to pro-inflammatory signals such as interferon-gamma (IFN-γ), TANs may survive longer than neutrophils circulating in the blood [30]. Neutrophil extracellular traps (NETs) consist of decondensed chromatin associated with neutrophil-derived proteins and can facilitate tumor cell adhesion, migration, invasion, and immune evasion. In OS, increased NET formation has been associated with metastatic phenotypes, and recent evidence suggests that NETs may contribute to the establishment of a pro-metastatic microenvironment. In addition, an emerging study indicated that AOC3 expression in OS can promote neutrophil recruitment, NET formation, tumor vascularization, and ultimately lung metastasis, further supporting a functional link between TANs, NETs, angiogenesis, and metastatic dissemination. Similar to the M1 and M2 polarization states of TAMs, Fridlender et al. demonstrated that TANs can also adopt either an anti-tumor N1 phenotype or a pro-tumor N2 phenotype [31]. TANs can also influence the tumor microenvironment through interactions with other immune cell populations [32]. Among the cell types most closely associated with TANs, there are myeloid-derived suppressor cells (MDSCs), which share a common myeloid origin. The N1 phenotype of TANs appears to be more common during the early phases of the disease and is associated with a more favorable treatment response and improved prognosis. In contrast, the N2 phenotype may contribute to the formation of an immunosuppressive TME, potentially reducing the effectiveness of immunotherapeutic approaches [33]. The distinctive functions of neutrophils in OS may be influenced by components of the bone matrix and by the specific cytokine environment within the OS, microenvironment, both of which can affect neutrophil polarization and activity [34]. Moreover, recent multi-omics studies have revealed several molecular subtypes of OS that differ in terms of prognosis and treatment response [35]. These subtypes also show distinct levels and patterns of neutrophil infiltration and activation, indicating that the mechanisms regulating neutrophil activity may differ among these subtypes. Within the OS microenvironment, TANs can directly promote tumor progression through the secretion of proteolytic and inflammatory mediators. For example, the TAN-derived matrix metalloproteinase-9 (MMP-9) has been associated with poor prognosis and may contribute to OS cell proliferation through the IRS-1/PI3K/AKT signaling axis. Moreover, single-cell analyses have identified distinct TAN-associated transcriptional programs linked to metastatic disease, implicating pathways such as HIF-1, PI3K/AKT, and JAK/STAT in TAN-mediated tumor progression. These observations suggest that TANs may actively shape the malignant phenotype rather than simply represent a consequence of tumor-associated inflammation [36,37].

#### 2.2.3. Myeloid-Derived Suppressor Cells

Myeloid-derived suppressor cells (MDSCs) are immature myeloid cells that have important functions within the TME. MDSCs are mainly divided into two groups according to their cellular lineage: polymorphonuclear MDSCs (PMN-MDSCs), derived from the granulocytic lineage, and monocytic MDSCs (M-MDSCs), derived from the monocytic lineage [38]. MDSCs commonly accumulate in pathological conditions characterized by persistent immune activation. Their development is associated with prolonged stimulation of myeloid cells in settings such as cancer, chronic infections, inflammatory conditions, and autoimmune diseases, where myeloid growth factors and inflammatory signals remain elevated over time. A key feature of MDSCs is their capacity to inhibit immune responses mediated by different immune cells, including T cells, B cells, and natural killer (NK) cells [39]. Both M-MDSCs and PMN-MDSCs exhibit several molecular characteristics involved in immunosuppression, such as increased activation of signal transducer and activator of transcription 3 (STAT3) and induction of endoplasmic reticulum stress. Interestingly, stimulated by the hypoxic microenvironment, MDSCs express high levels of vascular endothelial growth factor, vascular endothelial growth factor analog Bv8, basic fibroblast growth factor, and matrix metalloprotease 9 to facilitate angiogenesis and the formation of a pre-metastatic niche, which has a strong relationship with OS metastasis [40]. Moreover, MDSCs expressing high levels of IL-1β bind directly to OS cells through their interaction with the IL1R1 receptor, which is highly expressed in OS. This binding activates downstream nuclear factor kappa-light-chain-enhancer of activated B cells (NF-κB) signaling. NF-κB is recognized as a critical oncogene in OS, promoting cell proliferation, survival, migration, and invasion, while inhibiting apoptosis in 143B and MG63 human OS cells.

#### 2.2.4. Dendritic Cells

Dendritic cells (DCs) are key antigen-presenting cells that orchestrate T-cell-mediated antitumor responses, although they represent less than 5% of tumor-infiltrating myeloid cells in the OS tumor immune microenvironment. Conventional DCs (cDCs) in primary OS can be broadly classified into cDC1 and cDC2, both of which contribute to the regulation of antitumor immunity. Notably, cDC2 cells are enriched in metastatic lung lesions compared with primary and recurrent OS, suggesting a potential association with metastatic progression. Among DC subsets, CCR7^+^ DCs display enhanced migratory capacity toward lymphoid organs, where they can promote T-cell activation. Through downstream signaling pathways including PI3K-AKT, MAPKs, CCR7 regulates DC chemotaxis, migration, and cellular functions, suggesting that the CCR7 axis may contribute to immune-cell trafficking and metastatic behavior in osteosarcoma. Conversely, single-cell analyses have identified mature immunoregulatory dendritic cells (mregDCs) as an abundant DC population in OS with potential immunosuppressive activity [41]. mregDCs interact with T-regs through immunoregulatory pathways, including CD274–PDCD1 (PD-L1–PD-1) and PVR–TIGIT signaling. These interactions may promote T-reg recruitment and contribute to the establishment of an immunosuppressive tumor microenvironment. Similar mechanisms have been described in other tumor types, where mregDCs facilitate Treg accumulation through chemokine-dependent pathways, including the CCR4 and CXCL9/10–CXCR3 axes. Overall, these findings suggest that DCs have a dual role in OS, with distinct subsets potentially promoting antitumor immunity or, alternatively, supporting immune evasion, tumor progression, and metastasis [42].

#### 2.2.5. Natural Killer Cells

Natural killer (NK) cells are innate lymphoid cells with potent cytotoxic activity and the ability to control tumor growth and metastasis without prior antigen sensitization. Their antitumor effects are mediated through several mechanisms, including the release of cytotoxic granules containing perforin, granzymes, and granulysin, the production of cytokines such as IFN-γ and TNF-α, and the expression of death ligands, including Fas ligand (FasL) and TNF-related apoptosis-inducing ligand (TRAIL). In OS, NK cells represent a common tumor-infiltrating lymphocyte (TIL) population and can be further divided into distinct subclusters based on the expression of NK-associated markers such as NKG7 and GNLY. One subset co-expresses the T-cell markers CD3D and CD8A and is therefore classified as NKT cells. These cells display an activated cytotoxic phenotype, characterized by increased expression of GZMB, GZMA, and IFN-γ, suggesting active antitumor functions within OS lesions. In contrast, the conventional NK-cell subset shows limited expression of GZMB, IFN-γ, and PRF1, consistent with a relatively non-activated or functionally impaired state in the OS TME. Overall, these findings suggest that although NK cells are present within OS lesions, their antitumor activity may be heterogeneous, with NKT-like populations displaying greater cytotoxic activation than conventional NK cells [28].

#### 2.2.6. T-Lymphocytes

T cells are key mediators of adaptive immunity and play a central role in antitumor immune responses. In OS TME, T-cell infiltration is highly heterogeneous and varies according to tumor stage, anatomical site, and immune context. Tumor-infiltrating lymphocytes are predominantly localized in regions expressing human leukocyte antigen (HLA) class I, while CD4+ and CD8+ T cells are frequently enriched at the interface between pulmonary metastases and adjacent normal tissue [43]. Notably, metastatic lesions generally exhibit a higher density of T cells than primary or recurrent tumors, together with increased expression of immune checkpoint molecules, including PD-1, TIM-3, lymphocyte activation gene-3 (LAG-3), indoleamine 2,3-dioxygenase 1 (IDO1), and IFN-γ, reflecting a highly immunosuppressive microenvironment. Further evidence indicates that T-cell function in OS is tightly regulated through interactions with other immune cell populations. In particular, increased frequencies of TIM-3+PD-1− and TIM-3+ PD-1+ T cells have been detected within tumor tissues compared with peripheral blood, supporting the presence of local T-cell dysfunction and exhaustion [44]. Moreover, TAMs, particularly CD163+ macrophages, suppress T-cell proliferation and inflammatory cytokine production, whereas their depletion restores T-cell activity in vitro [44]. Although spontaneous tumor regression is extremely rare, isolated clinical observations have associated robust infiltration of activated cytotoxic T cells with favorable outcomes, further supporting the importance of T-cell-mediated immunity in OS [45]. Collectively, these findings highlight the complex and dynamic role of T cells in the OS TME and underscore their potential as therapeutic targets for immunomodulatory strategies.

### 2.3. Mesenchymal Stromal Cells

Mesenchymal stromal cells (MSCs) are non-hematopoietic progenitor stem cells and are regarded as possible precursor cells of OS cells [46]. Evidence suggests that MSCs function as intermediaries between OS tumor cells and the TME by releasing a variety of cytokines, chemokines, interleukins, and extracellular vesicles (EVs). Through paracrine interactions with OS cells, MSCs can influence several key features of tumor progression, including angiogenesis, cell proliferation, invasion, metastasis, immune regulation, and resistance to chemotherapy. MSCs can support OS growth, metastatic spread, and blood vessel formation through the secretion of different signaling molecules, such as chemokine ligand 5 (CCL5), stromal-derived factor 1 (SDF-1), CXCL12, CXCR4, IL-6, and vascular endothelial growth factor (VEGF) [46]. In addition, extracellular vesicles released by both OS cells and MSCs contribute to communication between cells by transferring also miRNAs/RNAs and proteins. In vitro studies have demonstrated that CXCR4 regulates the production VEGF by MSCs and surface MSCs can promote the growth of OS through VEGF-induced CXCR4 signaling. The positive expression of CXCR4/VEGF serves as an indicator of tumor metastasis and progression and is associated with a low survival rate [47]. IL-6 exhibits pro-tumorigenic activity in OS. It can enhance chemotherapy resistance, induce osteoclast activity at the tumor site, and enhance tumor cell invasion in OS. Notably, IL-6 is not secreted by OS cells but by cancer-activated stromal cells. It directly promotes tumor growth by activating the JAK/STAT3 pathway and influences prognosis [48].

### 2.4. Cancer Associated Fibroblasts

Cancer-associated fibroblasts (CAFs) are non stromal elements that play a pivotal role in chemioresistance in OS. They are abundantly present in both primary and metastatic OS lesions and are associated with unfavorable outcome and immunosuppressive microenvironment. In OS, cancer-associated fibroblasts (CAFs) contribute to immune evasion through several complementary mechanisms. CAF-derived CXCL12 interacts with CXCR4 on T cells, limiting their infiltration into the tumor and creating an “immune-excluded” zone around cancer cells [49]. CAFs also produce TGF-β, which suppresses T-cell proliferation, cytotoxic activity, and cytokine production while promoting the differentiation of CD4+ T cells into immunosuppressive regulatory T cells (Tregs), thereby supporting tumor progression and treatment resistance [50]. CAFs further promote immunosuppression by releasing chemokines and cytokines, including CCL2, IL-6, and CXCL8, which recruit immunosuppressive populations such as cells MDSCs, M2-polarized macrophages, and T-regs [51,52]. In particular, IL-6-mediated activation of the JAK/STAT3 pathway has been associated with increased immune suppression and OS progression [53]. In addition, CAF-driven remodeling of the ECM increases its density, creating a physical barrier that restricts the infiltration of cytotoxic T lymphocytes. Increased deposition of type I collagen and fibronectin, together with enhanced MMP activity, contributes to this immunosuppressive environment and may be associated with reduced responses to immune checkpoint inhibitors [54,55]. Overall, CAFs promote immune escape in OS by simultaneously suppressing T-cell activity, recruiting immunosuppressive cells, and remodeling the ECM.

### 2.5. Endothelial Cells and Pericytes

Endothelial cells (ECs) separate circulating blood from tissues and perform several functions, including maintaining blood fluidity, delivering nutrients and oxygen, removing metabolic waste, transporting immune cells, regulating immune responses, and forming new blood vessels [56]. OS requires a vascular supply for growth and waste removal and mainly obtains it through angiogenesis, which is stimulated by cytokines, chemokines, and growth factors released by the tumor microenvironment [57]. ECs may also undergo endothelial-to-mesenchymal transition (EndMT) and contribute to the formation of CAFs. CAF-derived SDF-1 can recruit ECs and promote angiogenesis, while IL-8 secretion by CAFs can stimulate neovascularization [56]. Pericytes are mesenchymal cells that surround small blood vessels and interact with ECs to regulate vascular stability and angiogenesis. ECs recruit pericytes through PDGF-B/PDGFR-β signaling, while other pathways, including Ang I and TGF-β, regulate endothelial viability and pericyte differentiation [58]. Although microvascular pericytes have been identified in OS specimens, their coverage has not been significantly correlated with tumor growth or metastasis [59].

### 2.6. Extracellular Vesicles

EVs are lipid-coated vesicles secreted by cells into the extracellular space, containing nucleic acids (DNA, RNA, and microRNAs), proteins, lipids (fatty acids and cholesterol), and intact organelles. In OS, EVs are representative special heterogeneous vesicles secreted by all cell types, produced by direct outward budding of the cell membrane [60]. Exosomes (30–150 nm) play an important role in the proliferation, metastasis, and drug resistance of OS. Immune effects of exosomes have also been observed in other tumors. For example, in some tumors, like breast cancer and mesethelioma, TGF-β1 in exosomes significantly downregulates CD8+ T cells and NK cells, inhibits lymphocyte activation, and further impedes tumor cell recognition [61]. Exosomes containing TGF-β1 have also been found to inhibit IL2-mediated lymphocyte proliferation more strongly than soluble TGF-β1 does, thereby increasing immune escape [62]. In breast cancer, TGF-β in exosomes induces monocyte differentiation and accumulation of immature MDSCs [63]. Exosomes produced by OS cells have been reported to have a bidirectional effect on tumors; however, there are only a few studies on their immune effects. Exosomes have tumor-associated antigens on their surfaces, which can interact with antigen-presenting cells and induce tumor-specific toxic T-cell immune responses [64]. Similar to exosomes secreted by osteoblasts, exosomes secreted by OS cells also have immunosuppressive effects, even at a much higher degree. Exosomes containing TGF-β, α-fetoprotein, and heat shock proteins downregulate T-cell proliferation, preventing effector T cells from working [65]. On the other hand, exosomes secreted by metastasized OS cells have been found to induce M2 polarization via TGF-β2, thus promoting further invasion and metastasis [66]. Exosomes can also disrupt the activity of NK cells and DCs via TIM-3 [67,68].

### 2.7. Immune Escape in OS

Despite the presence of immune cells within the OS TME, the antitumor immune response is frequently insufficient to achieve effective tumor control. One of the major mechanisms contributing to this phenomenon is the development of T-cell dysfunction and exhaustion [69]. Chronic exposure to tumor-associated antigens, together with persistent inflammatory and immunosuppressive signals, can induce a progressive loss of T-cell effector functions, characterized by impaired proliferation, reduced cytokine production, and diminished cytotoxic activity. Exhausted T cells typically exhibit sustained expression of inhibitory receptors, including programmed cell death protein 1 (PD-1), cytotoxic T-lymphocyte-associated protein 4 (CTLA-4), T-cell immunoglobulin and ITIM domain (TIGIT), lymphocyte activation gene-3 (LAG-3), and T-cell immunoglobulin and mucin-domain containing-3 (TIM-3) [70]. Importantly, the simultaneous expression of multiple inhibitory receptors is often associated with a more dysfunctional T-cell phenotype and may contribute to the limited ability of endogenous lymphocytes to eliminate malignant cells. The therapeutic rationale for immune checkpoint inhibitors (ICIs) is based on the possibility of restoring T-cell activity by interfering with inhibitory signaling pathways. However, clinical studies in OS have generally shown modest activity of single-agent checkpoint blockade, suggesting that PD-1/PD-L1 or other checkpoint pathways are unlikely to represent the only mechanisms responsible for immune suppression in this tumor. In addition, checkpoint expression alone does not necessarily indicate the presence of a functionally competent antitumor immune response. In an immunologically complex tumor such as OS, T-cell exhaustion may coexist with poor T-cell infiltration, inadequate antigen presentation, immunosuppressive myeloid populations, and metabolic restrictions, thereby limiting the capacity of checkpoint blockade to restore effective antitumor immunity. LAG-3 is frequently co-expressed with PD-1 on exhausted T cells and contributes to tumor-associated immune suppression. Its blockade can enhance T-cell proliferation and cytokine production. Given the high expression of LAG-3 in OS, combined targeting of LAG-3 and PD-1 represents a promising therapeutic approach. The combination of relatlimab (anti-LAG-3) and nivolumab (anti-PD-1) has shown encouraging antitumor activity in other solid tumors, while preclinical OS models suggest that dual blockade may enhance tumor regression. TIM-3 is an immune checkpoint associated with T-cell exhaustion and is also expressed by tumor-associated macrophages, contributing to an immunosuppressive microenvironment. Sabatolimab (anti-TIM-3), alone or combined with spartalizumab (anti-PD-1), has shown a favorable safety profile and preliminary antitumor activity in advanced solid tumors. However, evidence in OS remains limited, and further studies are needed to assess the therapeutic potential of TIM-3/PD-1 co-blockade [27,71]. Myeloid compartment represents a critical component of the OS TME and may substantially contribute to resistance to immunotherapy. TAMs, MDSCs, TANs, and other myeloid populations can establish an immunosuppressive environment that interferes with both innate and adaptive antitumor responses. Depending on the signals received from tumor cells and the surrounding stroma, macrophages can acquire phenotypes associated with tissue remodeling, angiogenesis, immune suppression, and tumor progression. In OS, the accumulation of immunosuppressive myeloid populations may promote tumor growth while simultaneously restricting effective T-cell activity. MDSCs are particularly relevant because of their ability to suppress T-cell proliferation and effector function through several complementary mechanisms. These include depletion of essential amino acids, production of reactive oxygen and nitrogen species, secretion of immunosuppressive cytokines, and modulation of antigen-presenting cells. Myeloid cells can also express inhibitory ligands and generate an environment that favors T-cell dysfunction. Consequently, even when ICIs successfully disrupt PD-1/PD-L1 signaling, the presence of a highly suppressive myeloid compartment may prevent T cells from regaining sufficient functional activity. This provides a potential explanation for why targeting a single immune checkpoint may be inadequate in OS [72].

On the other hand TAMs contribute to immunosuppression in OS by releasing cytokines such as IL-10 and TGF-β, which impair cytotoxic T-cell and NK-cell activity. Increased infiltration of CD163^+^ M2-like macrophages is associated with poor prognosis and enhanced metastatic potential. TAMs also promote immune evasion through PD-L1 upregulation, leading to T-cell exhaustion. Therefore, targeting TAMs, either through CSF1R inhibition or repolarization toward an M1-like phenotype using TLR agonists, represents a potential strategy to restore antitumor immunity in OS [73]. Therefore, the limited efficacy of ICIs in OS is likely to reflect the combined effects of multiple features of the TME rather than the activity of a single immunosuppressive pathway. In addition to T-cell exhaustion and myeloid-mediated suppression, the OS microenvironment is characterized by extensive stromal remodeling, abnormal vasculature, hypoxia, altered extracellular matrix composition, and metabolic stress. These factors can influence immune-cell recruitment, localization, activation, and survival, ultimately generating a spatially and functionally heterogeneous immune landscape [69]. Hypoxia represents an additional important component of this immunosuppressive milieu. Regions of low oxygen availability can promote tumor progression and alter the phenotype and function of infiltrating immune cells. Hypoxia-associated signaling may favor the accumulation or polarization of immunosuppressive myeloid populations while impairing T-cell effector functions. Similarly, metabolic competition between tumor and immune cells can restrict T-cell activity. High glucose consumption by tumor cells, accumulation of lactate, alterations in amino-acid availability, and changes in lipid metabolism may collectively generate metabolic conditions that are unfavorable for sustained T-cell activation. The extracellular matrix and stromal compartment may further contribute to immune exclusion [74]. Dense and aberrantly remodeled extracellular matrix components can interfere with immune-cell trafficking and limit the physical access of cytotoxic lymphocytes to tumor cells. In parallel, tumor-associated fibroblastic and stromal populations can produce cytokines, chemokines, and growth factors that promote tumor progression and immune suppression. Thus, the mere presence of immune cells within an OS lesion does not necessarily imply effective immune surveillance; their spatial distribution, functional state, and interaction with tumor and stromal compartments are equally important. Taken together, these observations suggest that resistance to ICIs in OS should be viewed as a multifactorial process involving the simultaneous presence of exhausted lymphocytes, suppressive myeloid populations, inadequate immune-cell recruitment, metabolic constraints, hypoxia, and stromal barriers.

## 3. 3D Modeling of the TME in OS: From Spheroids to Tumor Bioprinting

3D models provide a more physiologically relevant platform for investigating tumor microenvironment interactions than conventional two-dimensional (2D) in vitro assays. Although 2D culture systems have played a fundamental role in cancer research by advancing the understanding of protein expression, cellular biology, and the histological characteristics of various cancer subtypes, they present several important limitations. These include alterations in cell polarity and morphology, as well as disruption of cell–cell and cell–extracellular matrix interactions, which reduce their ability to accurately reproduce the in vivo microenvironment [75]. Consequently, there has been increasing interest in the development of 3D culture systems that more closely mimic physiological conditions. A wide range of 3D models has been established, each with distinct advantages and limitations. Depending on the preparation process, 3D models used in OS research can be generally summarized as spheroids, organoids, and bioprinting.

### 3.1. Spheroids

**Figure 2:**
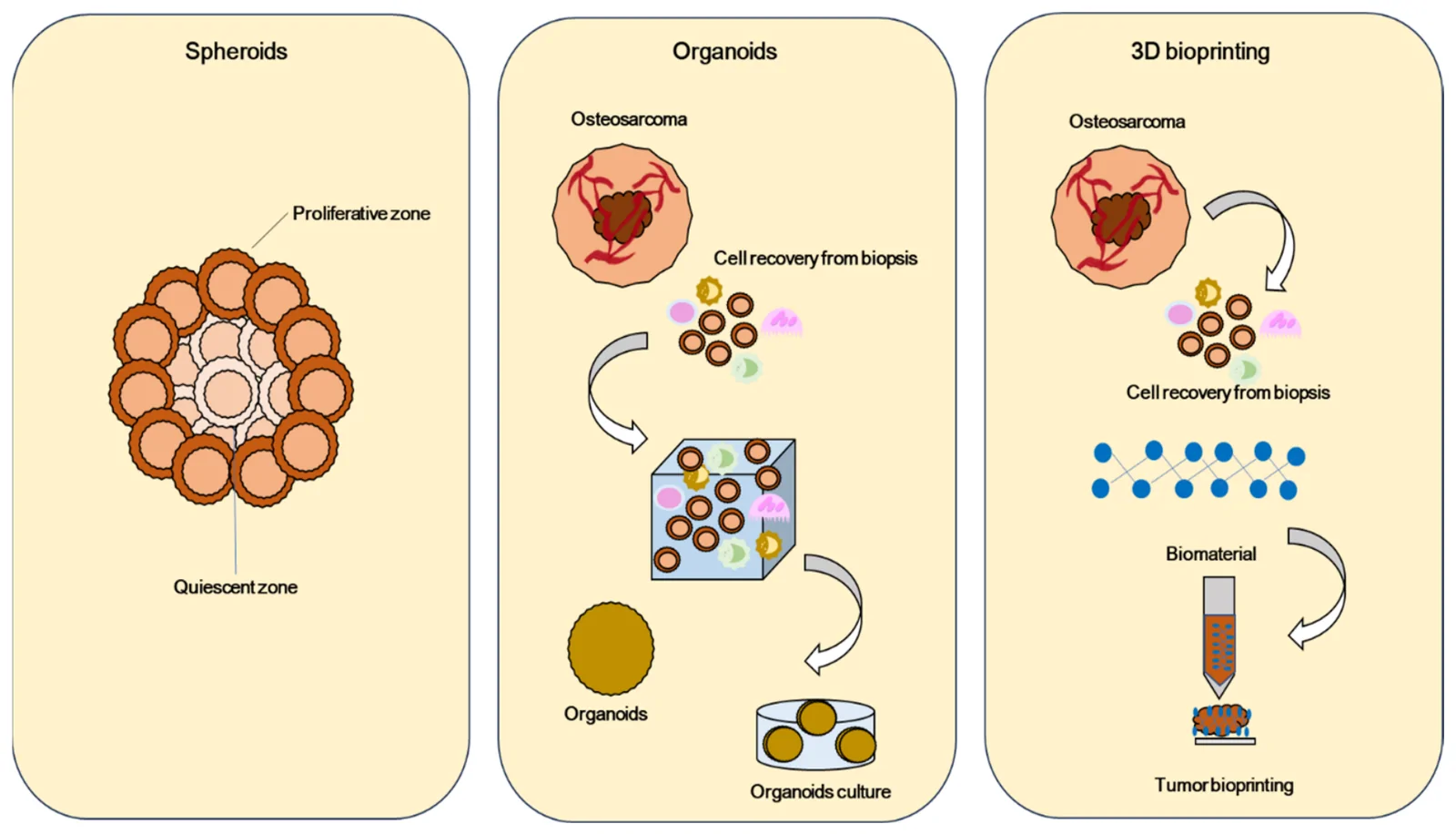
Overview of the principal three-dimensional (3D) culture platforms used to investigate the OS TME. Spheroids reproduce tumor architecture by generating proliferative and quiescent cellular regions. Patient-derived organoids are established from biopsy specimens and preserve the histological and molecular characteristics of the original tumor. Three-dimensional bioprinting combines patient-derived cells with biomaterials to generate engineered tumor constructs that closely recapitulate the native microenvironment, providing advanced platforms for disease modeling and preclinical drug testing.

Non-adherent tumor cell cultures, commonly referred to as 3D spheroids, are multicellular aggregates that self-assemble into compact, spherical structures primarily maintained by cell–cell interactions [76] (Figure 2). These models reproduce several key structural and functional features of solid tumors, including their 3D architecture, cellular heterogeneity, and the complex interplay between cells and the ECM. Compared with conventional 2D monolayer cultures, spheroids more closely mimic the biochemical and molecular characteristics of tumors, such as the secretion of soluble factors, gene expression profiles, and intracellular signaling networks. Furthermore, spheroids display enhanced metabolic features associated with tumor physiology, including increased reliance on anaerobic glycolysis, making them a more physiologically relevant platform for investigating cancer metabolism and its contribution to tumor progression [77]. Spheroids can be cultured in scaffold-free and scaffold-based ways.

#### 3.1.1. Scaffold-Free Spheroids

The architecture of tumor spheroids enables the establishment of physiologically relevant gradients of oxygen, nutrients, and metabolic waste, resulting in the formation of three concentric cellular regions: an outer proliferative layer, an intermediate zone characterized by hypoxia and reduced proliferation and an apoptotic zone [78]. This organization closely resembles that of solid tumors, making spheroids particularly valuable for investigating hypoxia-associated mechanisms of stress and metabolic adaptation. Considering their easy generation from both established cancer cell lines and patient-derived tumor samples, spheroids have become one of the most extensively used 3D culture systems for studying tumor biology, therapeutic resistance, and the preclinical evaluation of novel anticancer strategies (Figure 2). Based on their fabrication, spheroid can be categorized into scaffold-free and scaffold-based systems [79,80]. Scaffold-free spheroids are generated without the exogenous biomaterials or supporting matrices, relying only on spontaneous cell aggregation mediated by cell–cell adhesion while minimizing interactions with artificial substrates [81]. Several techniques have been developed to promote spheroid formation, with the hanging drop and ultra-low attachment (ULA) methods. In the first one, small droplets containing cell suspension are deposited on the underside of specialized culture plates. Gravity allow cells to accumulate at the bottom of each droplet, where they progressively establish intercellular contacts and self-organize into compact spheroids. This method allows the generation of highly reproducible spheroids with controlled size and morphology, making it particularly suitable for standardized experiments and high-throughput drug screening [82,83,84]. ULA plates are coated with hydrophilic polymers, which effectively prevent cell attachment to the culture surface [85,86]. Under these conditions, cells preferentially interact with surrounding cells rather than with the substrate, promoting spontaneous aggregation into multicellular spheroids. In OS, scaffold-free spheroids have become increasingly important because they better reproduce the reciprocal interactions between tumor cells and the ECM a critical regulator of tumor progression. ECM components actively contribute to cell invasion, metastatic dissemination, and resistance to chemotherapy [87,88]. For example, Cortini et al. demonstrated that OS spheroids generated using the hanging drop method preserved endogenous ECM deposition, which significantly affected the response of tumor cells to doxorubicin treatment [53]. The establishment of oxygen and nutrient gradients within OS spheroids also creates regions populated by metabolically stressed cells that undergo cell-cycle arrest or transition into a slow-proliferating, dormant phenotype. Since tumor dormancy is considered a major contributor to minimal residual disease and disease relapse, spheroids represent an excellent experimental platform for investigating these clinically relevant processes. Therefore, spheroids have the ability of 3D cultures to uncover molecular mechanisms that remain largely undetectable in conventional 2D systems [89,90]. In particular, scaffold-free spheroids have also been exploited to identify biomarkers associated with tumor aggressiveness and recurrence. Using MG-63-derived spheroids, it has been demonstrated that elevated expression of miR-664 was associated with increased invasive capacity, supporting its potential value as both a prognostic biomarker and a therapeutic target in OS [91]. These models are equally valuable for investigating cancer stem cell biology. Sphere-forming assays are routinely employed to evaluate the self-renewal potential, stemness properties, and drug resistance of OS stem-like cells. In this context, scaffold-free spheroids have also proven particularly useful for evaluating advanced therapeutic approaches, including nanoparticle-mediated drug delivery. Moreover, Wang et al. demonstrated that glutathione-scavenging nanoparticles efficiently eliminated chemotherapy-resistant OS stem-like cells within 3D spheroids [92]. Like other several studies, they have employed nanoparticles loaded with conventional chemotherapeutic agents, such as cisplatin, or functionalized with targeting peptides to investigate drug penetration, intratumoral distribution, and therapeutic efficacy under physiologically relevant conditions [93,94]. Natural compounds are another promising area of investigation using spheroid models, in particular on antiproliferative activity in MG63- and 143B-derived OS spheroids, demonstrating the suitability of 3D cultures as predictive preclinical platforms for evaluating the antitumor potential of bioactive molecules [95]. Finally, the biological complexity of spheroid models can be further increased by incorporating stromal and immune cell populations. Multicellular OS spheroids containing MSCs, monocytes, or additional non-malignant cell types provide a more precisely representation of the tumor microenvironment, enabling investigation of tumor–stroma interactions, immune modulation, and mechanisms that support tumor growth and progression in vivo [96].

#### 3.1.2. Scaffold-Based Spheroids

Scaffold-based 3D cultures rely on biomaterial-derived matrices that provide structural support while recreating key features of the ECM. In these models, tumor cells are either seeded onto preformed scaffolds or encapsulated within biomaterials, allowing them to adhere, proliferate, migrate, and organize into tissue-like architectures. Two main strategies are commonly employed to establish scaffold-based cultures. The first involves the use of acellular artificial scaffolds, including porous membranes, sponges, and culture inserts, which provide a 3D framework that supports cell attachment, migration, and proliferation while promoting the formation of spatially organized tissue-like structures [97]. The second approach consists of cell encapsulation within biomaterials, where tumor cells are suspended in hydrogel precursors before polymerization or cross-linking. Hydrogels such as gelatin methacryloyl (GelMA) and matrigel closely reproduce the hydrated and compliant nature of native ECM, thereby preserving cell viability, functionality, and physiological behavior [98,99,100,101]. In OS, scaffold-based models have enabled the development of increasingly biomimetic culture systems. Contessi Negrini et al. engineered a bone-inspired 3D model by fabricating scaffolds through 3D printing and enriching them with an extracellular matrix synthesized by osteogenically differentiated MSCs, for investigating tumor development and therapeutic responses within a bone-like microenvironment [102]. Consequently, scaffold-based systems have become valuable tools for examining how matrix stiffness, tensile loading, and shear stress regulate cancer stemness, invasive behavior, and metastatic dissemination [103]. The spheroid-containing constructs displayed significantly greater invasive capacity and increased resistance to chemotherapy, emphasizing the importance of multicellular organization in determining tumor aggressiveness and treatment response [104]. Recent technological advances have further improved the reproducibility and scalability of scaffold-based models. In particular, the integration of 3D-printed microwell platforms with low-adhesion culture techniques enables the generation of highly uniform spheroids while facilitating high-throughput screening of targeted therapies, biomaterials, and molecular interventions [105].

### 3.2. Organoids

Organoid cultures are 3D multicellular structures that arise from self-organizing stem cells derived from patient or mouse tumors [106]. These advanced 3D culture systems capable of reproducing key structural and functional features of their tissue of origin when maintained within supportive extracellular matrices, such as gelatin or collagen-based hydrogels. These models have emerged as powerful tools for investigating human tissue development, homeostasis, and disease mechanisms [107,108]. Tumor-derived organoids have been established from numerous cancers and are increasingly employed as clinically relevant platforms for studying tumor biology, therapeutic response, and personalized medicine. However, despite their demonstrated potential in several cancer types, the application of organoid technology in OS remains limited and lacks standardized protocols. This is mainly attributable to the distinctive biological complexity of OS, which differs substantially from the epithelial tumors that have traditionally driven organoid development. OS is characterized by a highly heterogeneous microenvironment composed of malignant osteoblastic cells, an extracellular osteoid matrix, and multiple non-tumoral cell populations. Osteoblast-like tumor cells produce osteoid, the non mineralized organic component of the bone matrix, while MSCs have been proposed as possible cells of origin. In addition, OS lesions contain osteoclast-like cells involved in bone remodeling and endothelial cells that contribute to tumor vascularization. Furthermore, the relatively low incidence of OS limits the availability of patient-derived specimens, while the small size of diagnostic biopsies and surgical samples often restricts the amount of tissue available for generating long-term cultures. Despite these limitations, recent studies have demonstrated the feasibility and potential value of OS-derived organoid models [109,110,111,112,113]. Beyond therapeutic testing, organoid systems may provide valuable opportunities for biomarker discovery [113]. More recently, the integration of organoid models with functional genomic approaches has further expanded their application in investigating mechanisms of treatment resistance. Xu et al. combined OS-derived organoid cultures with CRISPR-Cas9-based screening to identify genes involved in chemotherapy response. These findings highlight the ability of organoid-based platforms to uncover clinically relevant molecular mechanisms.

### 3.3. 3D Bioprinting to Model OS

The use of scaffolds in the fabrication of 3D in vitro models has led to the employment of 3D bioprinting in tumor research, an innovative technique initially developed and widely applied in regenerative medicine [114,115]. This advanced additive manufacturing approach utilizes both natural and synthetic biomaterials along with different cellular populations, to generate highly complex and reproducible experimental cancer models with well-defined architecture and composition. 3D bioprinting has recently emerged as an innovative approach for the generation of advanced in vitro tumor models capable of more accurately reproducing the structural and biological complexity of human cancers compared with conventional 2D cultures [116,117,118]. The ideal biomimetic ink for OS modeling should mimic the structural, physico-chemical, and biological properties of the ECM. Therefore, to study the mechanisms underlying tumorigenesis and cancer progression, inks must mimic the substrate on which the tumor develops and grows. On the other hand, they should reproduce ECM–cell interactions, allowing the study of the mechanisms occurring in osteolytic or osteoblastic lesions, both in primary and secondary bone tumors. The majority of the studies focus on hydroxyapatite (HA), as it more closely remembers the composition of bone. HA positively influences mechanical and biological properties of the constructs, including the extent of mineralization and collagen production exerted by host cells besides their osteogenic differentiation [119]. A physiologically relevant bioprinted model of bone cancer must reproduce not only the mineralized component of the tissue but also the complex vascular compartment. The presence of blood vessels is essential for recreating the dynamic interactions that occur within both healthy bone and the tumor microenvironment. In physiological conditions, the vascular system is closely involved in skeletal development, bone remodeling, and regeneration. In particular, it supports the exchange of nutrients, signaling molecules, and hematopoietic cells, contributes to the maintenance of specialized niches for hematopoietic stem cells in the bone marrow, and participates in the regulation of bone formation and homeostasis [120]. The role of the vasculature changes considerably in the tumor setting. As bone tumors develop, cancer cells actively modify the surrounding microenvironment to promote the formation and remodeling of blood vessels. This process supports tumor progression by increasing the availability of oxygen and nutrients and by creating routes through which malignant cells can enter the circulation and subsequently colonize distant tissues [121]. Angiogenic remodeling is generally stimulated by adverse conditions within the tumor microenvironment, including reduced oxygen availability and acidic extracellular pH. These factors disturb the physiological equilibrium between angiogenic activators and inhibitors, thereby promoting the expression of key pro-angiogenic mediators, particularly HIF and VEGF. While the tumor vasculature is mainly generated through the recruitment and remodeling of pre-existing vessels, other vascularization mechanisms may also contribute to its development. These include vasculogenesis, involving the formation of new vessels from vascular progenitor cells, whereby tumor cells themselves form vessel-like networks [122]. In order to reproduce the osteogenic and vasculogenic niches of bone in vascularized bone constructs, different approaches have been reported. In particular the employment of different combinations of composite materials like natural hydrogels mixed with polymers and bioceramic fillers and cell types like MSCs and human umbilical vein endothelial cells (HUVECs). Therefore a functional vascularized bone model should have a high mechanical stability and durability, specific biological features, and osteoconductive properties, which can be obtained by the combined use of rigid/synthetic polymers, natural hydrogels, and bioceramic fillers, respectively [123,124]. These requirements have shown promising results in the expression of osteogenic and angiogenic markers Furthermore, it has been reported that co-culturing HUVECs and MSCs promote cell proliferation and vascular network development [125,126]. Finally, dynamic perfusion culture through a bioreactor system can be beneficial for both bone and vascular regions, as the combination of liquid flux and mechanical cues (e.g., shear stress) enhance osteogenic differentiation, mineralization, and vascular endothelial growth factor (VEGF) expression. The here-reported strategies, though lacking in reproducing the complexity in the combination of the vascular and bone region, are promising for the development of the vascular network in 3D bone construction [127,128] (Table 1). Despite their potential to better reproduce the three-dimensional architecture and selected biochemical and mechanical features of the osteosarcoma microenvironment, 3D bioprinted models still have several limitations. Current models often include only a limited number of cell populations and therefore do not fully recapitulate the complexity of the osteosarcoma tumor microenvironment, particularly its immune, stromal, vascular, and bone-remodeling components. In addition, the use of established osteosarcoma cell lines may not adequately capture the molecular and phenotypic heterogeneity of patient tumors. Patient-derived cells may improve biological relevance, but their availability and expansion can limit reproducibility and scalability. Other challenges include the optimization of bioink composition and mechanical properties, the generation of functional vascular networks, and the lack of standardized bioprinting protocols. Thus, although 3D bioprinting represents a promising approach for modeling osteosarcoma and evaluating therapeutic responses, further optimization is required to achieve more physiologically relevant and reproducible models.

## 4. OS Patient Derived Xenograft

**Figure 3:**
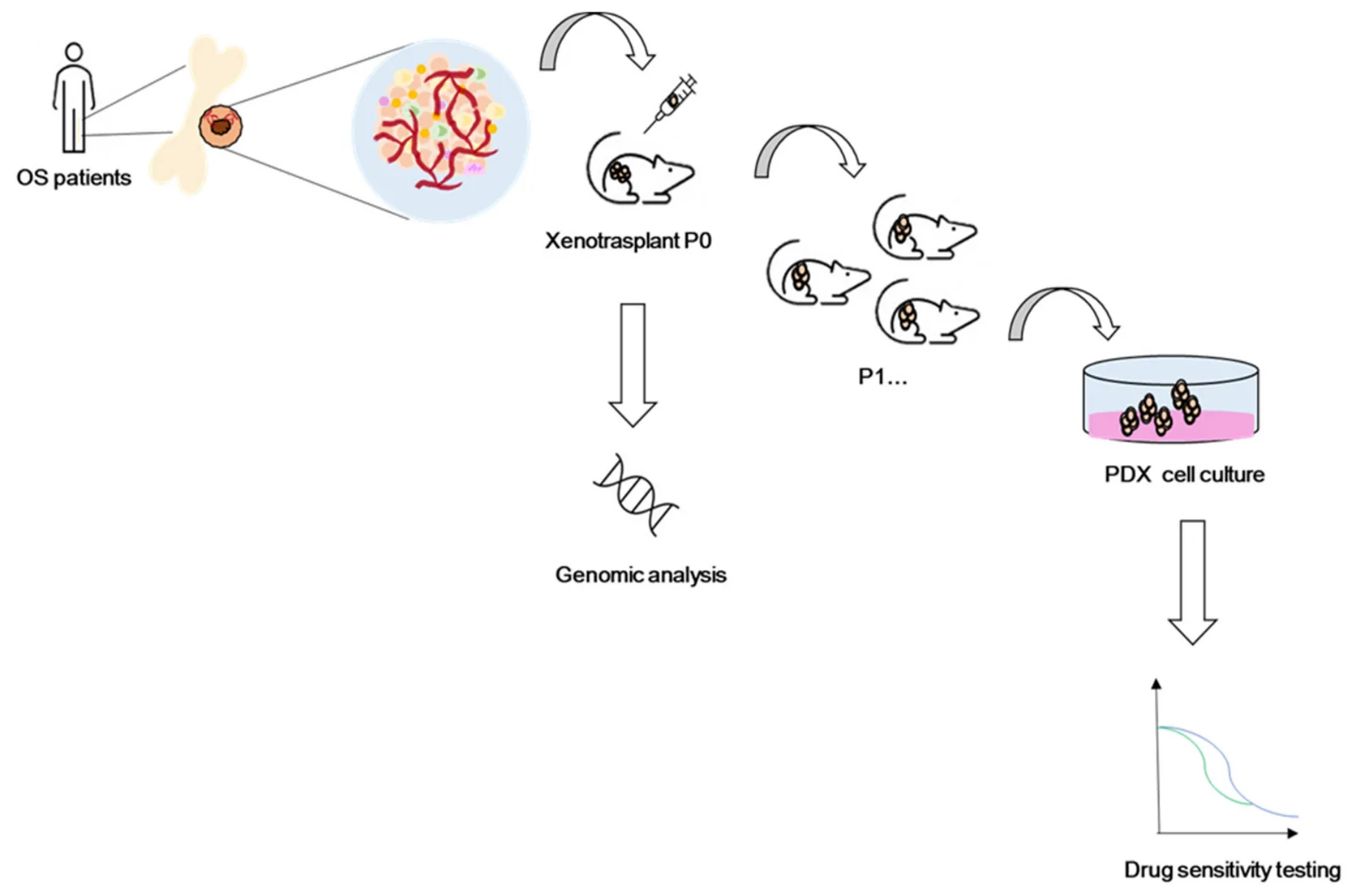
Schematic workflow for the establishment and application of OS patient-derived xenograft (PDX) models. Tumor specimens obtained from patients are implanted into immunodeficient mice to generate primary xenografts (P0), followed by serial passaging (P1 …) to maintain tumor propagation. PDX-derived tumor cells can then be used for in vitro culture, drug sensitivity testing, and genomic analyses.

PDX models are established by implanting freshly resected human tumor tissue directly into immunodeficient mice, thereby preserving many of the histological and molecular characteristics of the original tumor. In OS, tumor samples used for PDX generation can be obtained from biopsies, surgical resections of the primary lesion, following neoadjuvant chemotherapy, or metastases. These different tissue sources allow the establishment of models representing distinct stages of disease progression [128] (Figure 3). The reported success rate of OS PDX establishment, generally evaluated after at least three consecutive in vivo passages [129,130,131], varies considerably, ranging from approximately 20% to nearly 100%. Bioptic samples are typically limited in size but contain a high proportion of viable, treatment-naïve tumor cells, which may facilitate successful engraftment. In contrast, surgical specimens usually provide larger amounts of tissue; however, when collected after neoadjuvant chemotherapy they often contain extensive necrotic areas and therefore require careful pathological assessment to identify regions enriched in viable tumor cells. Interestingly, post-treatment samples may be enriched for chemoresistant and biologically aggressive tumor cell populations, a feature that has been associated with improved engraftment efficiency and a higher likelihood of establishing stable PDX models [132]. PDX models can be generated through ectopic or orthotopic implantation, depending on the anatomical site selected for tumor engraftment. In ectopic models, freshly tumor fragments are implanted into a subcutaneous in the flank of immunodeficient mice. An alternative implantation site is the interscapular brown adipose tissue, which has also proven suitable for subcutaneous tumor establishment. The main advantages of subcutaneous implantation include its technical simplicity and the ease with which tumor engraftment and growth can be monitored throughout the experiment [132]. Another ectopic approach involves implantation beneath the renal capsule, although this procedure is surgically more demanding [133,134]. To improve tissue viability and facilitate engraftment, tumor fragments are frequently embedded in matrigel before transplantation, providing additional extracellular matrix support and growth-promoting factors [135]. In OS, orthotopic xenografts are typically established by intrafemoral or intratibial implantation, allowing tumor growth within the native bone microenvironment. These models are considered particularly valuable because they better reproduce the interactions between tumor cells and the surrounding bone niche, which plays a crucial role in local disease progression and metastatic dissemination [136,137,138,139,140,141]. Nevertheless, some limitations should be considered. Maloney et al. demonstrated that intratibial inoculation of the murine OS cell line K7M2 into syngeneic BALB/c mice resulted in direct dissemination of tumor cells to the lungs, indicating that this approach may represent an experimentally induced rather than a truly spontaneous model of metastasis [140]. Furthermore, the establishment of intrafemoral or intratibial models often requires enzymatic dissociation of tumor specimens into single-cell suspensions before implantation. This process disrupts the original three-dimensional tumor architecture and may alter important cellular and microenvironmental interactions that are preserved when intact tumor fragments are transplanted [132].

### 4.1. Characterization of OS Patient-Derived Xenograft Models

Before PDX models can be reliably employed for preclinical investigations, they must undergo comprehensive histopathological, molecular, and genetic characterization to confirm their fidelity to the original patient tumor. To minimize the risk of cross-contamination, clonal selection, and genetic drift, the use of early-passage xenografts is generally recommended, together with continuous quality control throughout model propagation. Verification of sample identity is commonly performed using short tandem repeat (STR) profiling, which enables confirmation of the correspondence between the PDX and the donor patient [132,133,136]. Additional molecular characterization may include whole-genome sequencing, whole-exome sequencing, transcriptomic profiling, copy number alteration (CNA) analysis, structural variant detection, and mutation profiling. The genomic stability of PDX models has been extensively investigated across multiple tumor types [142]. Longitudinal analyses further revealed that genomic evolution occurs predominantly during the early in vivo passages, with CNA acquisition being more frequent between the first and fourth passages before progressively stabilizing as pre-existing tumor subclones become established. Comparable patterns of genomic evolution have also been described during the establishment of conventional human cancer cell lines in vitro [143]. Analyses of clonal composition indicated that OS PDXs retain the highest degree of clonal fidelity among several pediatric malignancies, including neuroblastoma and rhabdomyosarcoma, highlighting their ability to preserve the intratumoral heterogeneity of the original lesions [132]. Beyond genomic integrity, epigenetic stability also appears to be largely maintained during PDX propagation. In particular, there are only limited changes in DNA methylation profiles (approximately 1–6%) between primary OS specimens and the first corresponding PDX passage, with only minor additional variations observed in subsequent passages [142,143]. These findings support the use of OS PDX models for studies investigating epigenetic regulation and the evaluation of epigenetic therapies. Nevertheless, genetic and epigenetic alterations may gradually accumulate during serial transplantation and can influence biological pathways involved in treatment response, potentially modifying drug sensitivity compared with the original patient tumor [142]. These molecular changes are consistent with the shorter latency periods and accelerated tumor growth that are frequently observed in later PDX passages [132]. Taken together, these observations emphasize the importance of continuous molecular monitoring throughout PDX passages [142].

### 4.2. Limitations of PDX and Humanized PDX

Patient-derived xenograft (PDX) models have become valuable preclinical tools in osteosarcoma (OS) research because they can preserve several key characteristics of the original patient tumor, including histopathological features, genetic and epigenetic alterations, and intra- and inter-tumoral heterogeneity. In particular, orthotopic OS PDX models can provide relevant platforms for investigating tumor growth, metastatic dissemination, and therapeutic responses. However, conventional PDX models have important limitations that should be considered when studying the TME of OS. Since human tumor tissues are generally engrafted into immunodeficient mice to prevent rejection of the xenograft, these models lack a fully functional immune system and therefore cannot faithfully reproduce the interactions between osteosarcoma cells and human immune populations. This limitation is particularly relevant in OS, in which immune cells, stromal populations, osteoclasts, endothelial cells, and extracellular matrix components contribute to tumor progression, metastatic dissemination, and therapeutic resistance. Moreover, human stromal components, including cancer-associated fibroblasts, endothelial cells, and inflammatory cells, are progressively replaced by murine counterparts during PDX propagation, further limiting the ability of conventional PDX models to recapitulate the human TME. These limitations are particularly important when PDX models are used to evaluate immunotherapeutic strategies. Conventional immunodeficient PDX models are inherently unsuitable for investigating therapeutic mechanisms that require a functional human immune system, including immune checkpoint blockade and other immune cell-based approaches. Furthermore, differences between the human and murine stromal, vascular, cytokine, and immune compartments may influence tumor behavior and therapeutic responses. In OS, these limitations are further compounded by the unique characteristics of the bone microenvironment, which cannot be completely reproduced in a murine host. Humanized PDX models have therefore emerged as a promising strategy to partially overcome these limitations. These models combine patient-derived tumors with a human immune system reconstituted in immunodeficient mice, thereby providing an experimental platform in which patient-derived tumor features can be investigated in the presence of human immune cells. Humanization can be achieved through different approaches, including the transplantation of human CD34+ hematopoietic stem and progenitor cells or the adoptive transfer of human peripheral blood mononuclear cells. In the context of OS, humanized mouse models have already been used to investigate the contribution of human immune cells to tumor growth and to evaluate responses to immune checkpoint inhibition. For example, CD34+ hematopoietic stem cell-engrafted NSG mice bearing xenografts have demonstrated human immune-cell reconstitution and have provided evidence that the degree of human immune-cell engraftment can influence tumor growth. Other humanized OS models have demonstrated increased CD4+ and CD8+ T-cell infiltration and reduced pulmonary metastatic burden following treatment with the PD-1 inhibitor nivolumab, highlighting their potential utility for studying tumor–immune interactions and immunotherapy. Importantly, humanized PDX models should not be considered a complete recapitulation of the human OS TME [144]. Human immune-cell reconstitution may be incomplete or heterogeneous, and species-specific differences in cytokines, chemokines, stromal cells, extracellular matrices, and bone biology remain significant challenges. Moreover, humanization increases the technical complexity, cost, and experimental time required to establish these models. In particular, peripheral blood mononuclear cell-based models may be limited by xenogeneic graft-versus-host disease, whereas CD34+ hematopoietic stem cell-based approaches require longer periods for immune reconstitution and may not generate all human immune-cell populations at physiological levels. Thus, humanized PDX models may represent an important intermediate platform between conventional PDX models, which are highly valuable for studying patient-specific tumor biology and drug responses, and more complex experimental systems specifically designed to investigate human tumor–immune interactions. Their integration with orthotopic implantation and advanced approaches to model the bone microenvironment may be particularly valuable for improving the preclinical evaluation of immunotherapeutic strategies in OS [142].

## 5. Lung Metastatic Microenvironment

The pulmonary metastatic microenvironment deserves dedicated consideration because the lung represents the most clinically relevant site of osteosarcoma metastasis and provides a distinct cellular and extracellular context that can support metastatic colonization and outgrowth [1,2]. Rather than functioning as a passive recipient of disseminated tumor cells, the lung constitutes a dynamic microenvironment in which metastatic OS cells interact with resident and recruited immune cells, endothelial cells, fibroblasts, the ECM, and other stromal populations. These interactions can influence the survival of circulating tumor cells, their extravasation into the pulmonary parenchyma, establishment of premetastatic niches, immune evasion, angiogenesis, and subsequent metastatic expansion [1,2,3].

### 5.1. Lung Macrophages

Lung macrophage are important components of the metastatic niche. Alveolar macrophages and recruited monocyte-derived macrophages can respond to tumor-derived signals and, depending on their functional state, exert either antitumor or tumor-supportive effects. In the metastatic setting, tumor-associated macrophages may promote osteosarcoma-cell survival and proliferation through paracrine signaling, facilitate tissue remodeling, and contribute to an immunosuppressive milieu [145,146]. In particular, osteosarcoma-derived CCL2 has been shown to promote the accumulation of tumor-associated macrophages in the lung, while inhibition of the CCL2 axis reduced pulmonary metastatic burden in experimental models [146]. These findings support a role for macrophage recruitment and functional reprogramming in the establishment of the pulmonary metastatic niche. Importantly, macrophage function should not be viewed simply through a binary M1/M2 framework, as pulmonary macrophages exhibit substantial phenotypic and functional heterogeneity that is shaped by local signals and disease stage [145,147].

### 5.2. Neutrophils

Neutrophils represent another important component of the pulmonary metastatic niche. Tumor-associated neutrophils can influence metastatic colonization through the release of proteases, reactive oxygen species, cytokines, chemokines, and neutrophil extracellular traps (NETs) [148,148]. In OS, tumor-derived factors have been shown to promote neutrophil recruitment to the lung and contribute to the formation of a premetastatic microenvironment. In particular, AOC3 has been implicated in osteosarcoma lung metastasis through the recruitment of tumor-associated neutrophils, NET formation, and modulation of tumor vascularization [149]. Similarly, osteosarcoma-derived secreted factors can induce neutrophil infiltration and pulmonary stromal remodeling before the establishment of overt metastatic lesions [148]. These observations suggest that neutrophils may contribute not only to the later stages of metastatic growth but also to the preparation of the lung for tumor-cell colonization.

### 5.3. Lung Endothelium

The lung endothelium is particularly relevant during the transition from circulating tumor cells to established metastatic lesions. Endothelial cells regulate tumor-cell arrest, adhesion, and transendothelial migration and can respond to tumor-derived angiogenic and inflammatory signals [145,150]. Once metastatic lesions become established, endothelial remodeling and angiogenesis can provide osteosarcoma cells with access to oxygen and nutrients while also creating additional opportunities for interaction with circulating immune cells [145,149]. Thus, endothelial cells may contribute both to the initial seeding process and to the subsequent growth of pulmonary metastases.

### 5.4. Fibroblasts and Other Mesenchymal Stromal Cells

These cells can further modify the metastatic niche. Activated fibroblasts may produce ECM proteins, matrix-remodeling enzymes, chemokines, and growth factors that alter the physical and biochemical properties of the lung [145,148]. Notably, osteosarcoma-derived secreted factors have been reported to induce fibroblast activation and promote pulmonary deposition of extracellular matrix components, including fibronectin and collagen, thereby contributing to the formation of a permissive premetastatic niche [148]. Fibroblast-derived signals may additionally interact with immune and endothelial compartments, highlighting the interconnected nature of the pulmonary metastatic microenvironment.

### 5.5. ECM

The ECM should therefore be considered an active regulator rather than merely a structural scaffold. Alterations in collagen, fibronectin, laminins, proteoglycans, and matrix-associated signaling molecules can affect tumor-cell adhesion, migration, proliferation, and mechanotransduction [8]. Matrix remodeling can also generate biochemical signals that influence both tumor and stromal cells. In osteosarcoma, changes in ECM composition and activity of matrix-remodeling enzymes have been associated with tumor progression and metastatic behavior [8]. In the lung, osteosarcoma-derived factors may further promote ECM deposition and remodeling, potentially facilitating subsequent tumor-cell colonization [148]. Finally, these individual components operate as an interconnected network rather than as independent cellular compartments. Osteosarcoma cells can release cytokines, chemokines, extracellular vesicles, and other factors that recruit and reprogram pulmonary stromal and immune cells, while macrophages, neutrophils, endothelial cells, and fibroblasts provide reciprocal signals that influence tumor-cell behavior [145,147,148,150]. Single-cell analyses of osteosarcoma lung metastases have further demonstrated the cellular heterogeneity of this immunosuppressive microenvironment, supporting the concept that metastatic progression depends on coordinated interactions among multiple immune and stromal populations [147]. Consequently, pulmonary metastasis is best understood as an ecological process involving continuous communication between malignant cells and their surrounding niche. A more detailed characterization of these cellular interactions, including their spatial organization and temporal evolution, may identify microenvironmental dependencies that distinguish metastatic osteosarcoma from the primary tumor and could reveal therapeutic opportunities for preventing or treating pulmonary metastatic disease [145,147].

## 6. Conclusions

**Figure 4:**
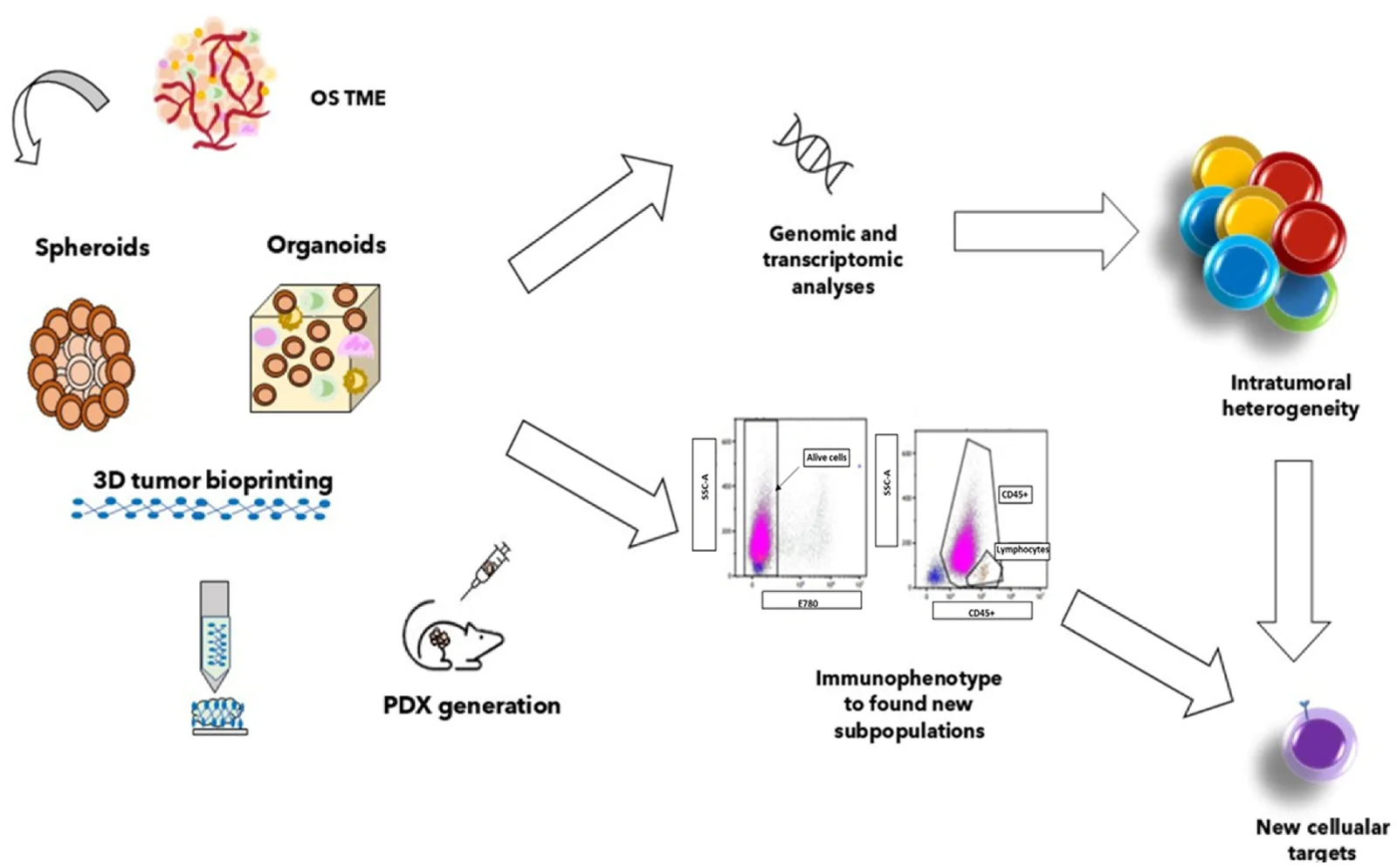
Tumor microenvironment, experimental models and potential therapeutic targets.

OS remains one of the most aggressive primary bone malignancies, with limited therapeutic progress over the past decades and poor outcomes for patients with metastatic or recurrent disease [1]. Increasing evidence indicates that tumor progression is not exclusively driven by the intrinsic genetic alterations of malignant cells but also by the complex interactions established within the TME. Bone-resident cells, immune populations, extracellular matrix components, soluble mediators, hypoxia, and extracellular vesicles cooperate to create a highly dynamic and immunosuppressive niche that promotes tumor growth, metastatic dissemination, and resistance to conventional therapies. Understanding the reciprocal communication among these components is therefore essential for identifying novel biomarkers and therapeutic targets capable of overcoming the current limitations of OS treatment. Among the cellular components of the TME, osteoblasts, osteoclasts, mesenchymal stromal cells, and immune cells actively regulate bone remodeling, immune escape, angiogenesis, and extracellular matrix remodeling. At the same time, acellular elements, including cytokines, chemokines, hypoxia, and extracellular vesicles, orchestrate molecular signaling networks that further sustain tumor progression [11,75]. These findings support the concept that OS should be considered not only as a disease of malignant osteoblastic cells but as a complex tissue ecosystem in which multiple cellular and molecular players contribute to disease evolution. The growing understanding of OS biology has also highlighted the limitations of conventional 2D in vitro models, which fail to reproduce the structural, cellular, and biochemical complexity of the native tumor microenvironment. Consequently, advanced experimental platforms, including multicellular spheroids, scaffold-based cultures, patient-derived organoids, 3D bioprinted constructs [78,122], and PDXs [131,143], have emerged as valuable complementary tools for investigating tumor biology under more physiologically relevant conditions. Although each model presents specific advantages and limitations, their combined application provides increasingly accurate systems for studying TME interactions, therapeutic resistance, biomarker discovery, and the preclinical evaluation of novel treatment strategies. In future advances in OS, research will likely rely on the integration of these next-generation experimental models with high-resolution molecular technologies, including single-cell, multi-omics approaches, and patient-derived immune-competent platforms. Such multidisciplinary strategies will improve our understanding of tumor heterogeneity and the dynamic evolution of the TME, facilitating the identification of actionable therapeutic vulnerabilities. Overall, a more comprehensive characterization of the OS TME, together with the development of increasingly biomimetic and standardized preclinical models, will be essential for accelerating translational research and advancing precision medicine approaches. These efforts hold considerable promise for improving patient stratification, identifying more effective therapeutic combinations, and ultimately enhancing the clinical management and survival of patients with OS (Figure 4).

### Translational Implications and Future Perspectives

Among the currently available 3D platforms, multicellular spheroids represent one of the most accessible and experimentally established approaches for modeling relevant features of OS biology. Spheroids can reproduce several characteristics that are poorly captured by monolayer cultures, including cell–cell interactions, spatial gradients of oxygen and nutrients, extracellular matrix deposition, proliferative heterogeneity, and reduced drug penetration. These features make spheroid-based systems particularly useful for investigating tumor growth, invasion, therapeutic response, and mechanisms of drug resistance. Their relatively simple generation and scalability also facilitate their potential application in medium- to high-throughput drug screening. Nevertheless, conventional spheroids remain limited by their simplified cellular composition and by the absence of several critical components of the bone TME, including vascular, immune, and mechanically active compartments. Patient-derived organoid models represent a further step toward personalized OS research. By preserving, to varying degrees, the cellular and molecular characteristics of the original tumor, organoid systems provide an opportunity to investigate inter-patient heterogeneity and to evaluate differential responses to therapeutic agents ex vivo. In this context, organoids may become particularly relevant for the identification of patient-specific vulnerabilities and for the preclinical evaluation of drug combinations. However, their translational potential should currently be interpreted with caution. Although organoid-based approaches have provided valuable evidence in OS research, their routine use for clinical decision-making has not yet been prospectively validated. In particular, differences in culture conditions, extracellular matrix composition, tissue processing, cellular composition, and the extent to which the original TME is retained can influence organoid phenotype and therapeutic response. Standardization and prospective correlation with patient outcomes will therefore be essential before organoid-guided treatment selection can be incorporated into clinical practice. Three-dimensional bioprinting represents a more recent and technically advanced strategy with substantial potential for OS. Unlike conventional spheroids and organoids, bioprinting allows the spatial organization of multiple cell types and biomaterials with greater control over tissue architecture. This is particularly relevant to OS because the tumor develops within a mineralized and mechanically complex bone environment [116]. Bioprinted constructs can therefore be designed to incorporate tumor cells together with osteoblasts, mesenchymal stromal cells, endothelial cells, immune populations, and bone-mimetic extracellular matrices, potentially providing more physiologically relevant models of tumor–stroma interactions, invasion, angiogenesis, and therapeutic response. Despite these advantages, bioprinting should currently be regarded primarily as an emerging preclinical technology in OS rather than a clinically validated platform. Major challenges include reproducibility, bioink standardization, control of cellular organization, vascularization, long-term stability, scalability, and the development of standardized protocols that allow meaningful comparison across laboratories. Its future translational relevance will therefore depend not only on increasing biological complexity but also on demonstrating that this complexity improves the prediction of therapeutic responses compared with simpler and more established models. Patient-derived xenografts provide an additional complementary approach by preserving several features of the original tumor within an in vivo context and have contributed substantially to the study of OS growth, metastatic behavior, and therapeutic response [128]. Nevertheless, conventional PDX models are constrained by the replacement or absence of the human immune system and by the interaction of human tumor cells with a murine stromal environment. These limitations are particularly relevant when studying immunotherapies and human-specific tumor–immune interactions. Thus, while PDX models remain valuable for in vivo validation, their integration with more physiologically representative human 3D systems may provide a more comprehensive translational framework. A major future direction will be the convergence of these complementary platforms rather than the replacement of one model by another. In particular, the integration of patient-derived spheroids or organoids with stromal, vascular, and immune components could generate increasingly representative models of the OS. Similarly, incorporating patient-derived organoids into bone-mimetic or bioprinted matrices may combine the biological heterogeneity preserved by organoid systems with the spatial and mechanical control provided by tissue engineering approaches. Such hybrid platforms could be particularly valuable for investigating processes that cannot be adequately reproduced by isolated tumor-cell cultures, including metastatic dissemination, osteogenic interactions, extracellular matrix remodeling, immune evasion, and treatment-induced changes in the TME. The incorporation of high-resolution molecular technologies will further increase the translational potential of these models. Single-cell RNA sequencing and spatially resolved approaches can characterize cellular heterogeneity and identify rare tumor or stromal populations that may contribute to disease progression or therapeutic resistance. Integration with transcriptomic, epigenomic, proteomic, metabolomic, and other multi-omics datasets may provide a more comprehensive representation of the molecular networks connecting malignant cells with their microenvironment. Importantly, these technologies could enable the identification of reproducible molecular signatures associated with treatment response, metastatic potential, or resistance, while also providing mechanistic explanations for phenotypes observed in experimental models. In the longer term, combining patient-derived 3D models with single-cell and spatial multi-omics may allow the functional characterization of individual patient tumors at unprecedented resolution. Another important future perspective concerns the development of immune-competent and patient-derived models. Because immune suppression and tumor–immune interactions are increasingly recognized as important determinants of OS progression and therapeutic response, models lacking relevant immune components may provide an incomplete representation of the disease. The development of platforms incorporating autologous or physiologically relevant immune cells, together with tumor and stromal compartments, could facilitate the investigation of immune-mediated mechanisms and the rational testing of immunotherapeutic combinations. However, these approaches remain technically challenging and require further validation before they can be considered reliable predictors of clinical response. From a translational perspective, the principal challenge is therefore not simply to develop increasingly complex models, but to establish which degree of complexity provides clinically meaningful information. A useful future framework may progress from relatively standardized spheroid-based assays for high-throughput screening, to patient-derived organoids for individualized assessment of tumor biology and drug response, and ultimately to more complex organoid–stroma, organ-on-chip, or bioprinted systems when specific biological questions require the reconstruction of vascular, immune, mechanical, or spatial features of the TME. This model hierarchy could be complemented by PDX or other in vivo systems for subsequent validation of the most promising therapeutic findings. Several barriers must nevertheless be overcome before these approaches can be routinely translated into clinical practice. These include the lack of standardized protocols, inter-laboratory variability, limited sample availability, differences in tissue processing and culture conditions, incomplete representation of the native TME, and the absence of universally accepted criteria for assessing model fidelity. Importantly, prospective studies correlating ex vivo responses with clinical outcomes are needed to determine whether these models can reliably predict treatment efficacy. Regulatory and logistical considerations, including reproducibility, scalability, cost, turnaround time, and quality control, will also influence their eventual integration into diagnostic and therapeutic workflows. Overall, the translational future of OS modeling will likely depend on the complementary integration of experimentally established 3D platforms with increasingly sophisticated patient-specific technologies. Spheroids and organoid-based systems currently provide a relatively accessible and biologically informative foundation for studying tumor heterogeneity and therapeutic response, whereas bioprinting, immune-competent models, organ-on-chip systems, and integrated multi-omics platforms represent promising emerging technologies that remain to be more extensively validated in OS. Rather than pursuing complexity for its own sake, future research should focus on determining whether each technological advance provides measurable improvements in biological fidelity and, most importantly, in the prediction of patient outcomes. The ultimate goal will be to establish standardized, reproducible, and clinically informative platforms capable of bridging the gap between experimental OS, research and precision oncology, thereby facilitating patient stratification, the identification of actionable vulnerabilities, and the development of more effective therapeutic combinations.

## Figures and Tables

**Table 1:** Schematic representation of commonly used in vitro and in vivo models, including scaffold-free spheroids, scaffold-based spheroids, organoids, 3D bioprinting, and patient-derived xenografts (PDXs). The different models recapitulate distinct features of OS biology and provide complementary platforms for investigating tumor heterogeneity, the tumor microenvironment, cell–matrix interactions, spatial organization, and therapeutic response.

Model	Key Features	Advantages	Limitations	Main Application	References
Scaffold-free spheroids	3D aggregation of OS cells under non-adherent conditions, generating compact cellular aggregates.	Simple, inexpensive and relatively rapid; for high-throughput screening; reproduces cell–cell interactions.	Limited ECM; simplified cellular composition; limited architectural control; tumor heterogeneity may be poorly represented.	Drug screening; proliferation and invasion; chemotherapy and radiotherapy response.	[78,79,80]
Scaffold-based spheroids	OS spheroids embedded within or generated inside a 3D biomaterial matrix.	Provides additional ECM, mechanical and biochemical cues. Improves biomimicry and enables investigation of cell–ECM interactions. Scaffolds can be designed to reproduce aspects of the bone microenvironment using collagen, hydroxyapatite, GelMA and other biomaterials.	More expensive and technically demanding than scaffold-free spheroids. Scaffold properties can strongly influence cell behavior and drug response. Lower standardization and more difficult downstream analysis.	Drug screening, invasion studies, mineralization and investigation of tumor–bone interactions.	[97,98]
Organoids	Self-organizing 3D structures generated from OS cells or patient-derived tumor tissue, often maintained within an ECM-rich matrix.	High patient specificity and potential to preserve aspects of tumor heterogeneity and molecular characteristics.	Tumor heterogeneity, patient-specific characteristics, cell–cell interactions and aspects of tumor organization.	Precision oncology, individualized drug testing and investigation of tumor biology.	[107]
3D bioprinting	Layer-by-layer deposition of cells and biomaterials or bioinks according to a predefined digital architecture.	Provides a high degree of spatial and architectural control. Geometry, porosity, stiffness and cellular distribution can be precisely modulated. Allows incorporation of multiple cell types and bone-like biomaterials	High cost and technical complexity. Requires specialized equipment and expertise. Bioink composition and printing conditions can affect cell viability and phenotype. Limited throughput and standardization.	Spatial organization, ECM, bone-like microenvironment, tumor–stroma interactions architecture. Advanced drug screening, tumor invasion, angiogenesis.	[116,118]
PDXs	Implantation of fresh patient-derived OS tissue or tumor fragments into immunodeficient mice, followed by in vivo tumor growth and, in some serial transplantation.	High translational relevance. Can preserve tumor architecture, heterogeneity and patient-specific characteristics. Provides an in vivo context for tumor growth, invasion, metastasis and treatment response. Useful for validation of therapeutic targets and biomarkers.	Expensive, time-consuming and low-throughput. Requires animal facilities and specialized expertise. Immunodeficient hosts lack a functional human immune system. Human stromal components can progressively be replaced by murine stroma during serial passaging. Engraftment is not always successful.	Tumor growth, tumor architecture, invasion, metastatic behavior, pharmacological response and patient-specific tumor characteristics. Drug validation, biomarker studies, metastatic studies and translational/precision oncology.	[128,129]

## Data Availability

No new data were created or analyzed in this study. Data sharing is not applicable to this article.

## Abbreviations

The following abbreviations are used in this manuscript:

OS	Osteosarcoma
TME	Tumor microenvironment
3D	Three-dimensional
PDXs	Patient-derived xenografts
T-reg	T regulatory cell
ECM	Extracellular matrix
EVs	Extracellular vesicles
MMPs	Matrix metalloproteinases
TAMs	Tumor-associated macrophages
TANs	Tumor-associated neutrophils
MDSCs	Myeloid-derived suppressor cells
ECs	Endothelial cells
CAFs	Cancer associated fibroblasts
DCs	Dendritic cells
MSCs	Mesenchymal stem cells
CTCs	Circulating tumor cells
RUNX2	RUNX family transcription factor 2
IL-6	Interleukin 6
LAG-3	Lymphocyte activation gene-3
IDO1	Indoleamine 2,3-dioxygenase-1
2D	Two-dimensional
ULA	Ultra-low attachment
GelMA	Gelatin methacryloyl
HA	Hydroxyapatite
HUVECs	Human umbilical vein endothelial cells
CNA	Copy number alteration
